# scMatch: a single-cell gene expression profile annotation tool using reference datasets

**DOI:** 10.1093/bioinformatics/btz292

**Published:** 2019-04-26

**Authors:** Rui Hou, Elena Denisenko, Alistair R R Forrest

**Affiliations:** Harry Perkins Institute of Medical Research, QEII Medical Centre and Centre for Medical Research, The University of Western Australia, Nedlands, Perth, WA 6009, Australia

## Abstract

**Motivation:**

Single-cell RNA sequencing (scRNA-seq) measures gene expression at the resolution of individual cells. Massively multiplexed single-cell profiling has enabled large-scale transcriptional analyses of thousands of cells in complex tissues. In most cases, the true identity of individual cells is unknown and needs to be inferred from the transcriptomic data. Existing methods typically cluster (group) cells based on similarities of their gene expression profiles and assign the same identity to all cells within each cluster using the averaged expression levels. However, scRNA-seq experiments typically produce low-coverage sequencing data for each cell, which hinders the clustering process.

**Results:**

We introduce scMatch, which directly annotates single cells by identifying their closest match in large reference datasets. We used this strategy to annotate various single-cell datasets and evaluated the impacts of sequencing depth, similarity metric and reference datasets. We found that scMatch can rapidly and robustly annotate single cells with comparable accuracy to another recent cell annotation tool (SingleR), but that it is quicker and can handle larger reference datasets. We demonstrate how scMatch can handle large customized reference gene expression profiles that combine data from multiple sources, thus empowering researchers to identify cell populations in any complex tissue with the desired precision.

**Availability and implementation:**

scMatch (Python code) and the FANTOM5 reference dataset are freely available to the research community here https://github.com/forrest-lab/scMatch.

**Supplementary information:**

Supplementary data are available at *Bioinformatics* online.

## 1 Introduction

Although the whole-transcriptome analysis of single cells has been possible since 2009 (Tang *et al.*, 2009) only recently has it become broadly applied in the research community. This is due to the development of new massively multiplexed single-cell RNA sequencing (scRNA-seq) protocols (Han *et al.*, 2018; Hashimshony *et al.*, 2012; Macosko *et al.*, 2015; Picelli *et al.*, 2013; Rosenberg *et al.*, 2018) and the broad availability of commercial platforms for generating these libraries. Barcode-based tracking methods (molecular-, cellular- and plate-level tags) now allow us to profile gene expression in thousands of cells. This advance in single cell profiling is enabling characterization of the diverse cell types that make up various tissues (Regev *et al.*, 2018) and to study biological processes, such as cell development (Bendall *et al.*, 2014; Klein *et al.*, 2015; Setty *et al.*, 2016; Trapnell *et al.*, 2014), cell state transition (da Rocha *et al.*, 2018; Haghverdi *et al.*, 2016; Shin *et al.*, 2015; Treutlein *et al.*, 2014) and multi-cellular interactions (Tay *et al.*, 2010; Thompson *et al.*, 2014; Wang *et al.*, 2014).

For the majority of these new studies, high cell count, low-sequencing depth strategies are being used; however, low-sequencing depth scRNA-seq data typically only measures the expression of the most highly expressed 500–2000 genes per cell (Macosko *et al.*, 2015; Zheng *et al.* 2017). Additionally, cells have different RNA complexities, e.g. embryonic stem cell transcriptomes are more complex (expressing a broad range of genes) than fully differentiated cells which have transcriptomes more skewed to high expression of a smaller subset of genes. This translates to variable numbers of genes detected per cell and consequently variable numbers of ‘dropouts’ (genes that are expressed but not detected) for different cell lineages.

To date, most publications analysing scRNA-seq data start by unsupervised clustering of the cells based on similarity between their gene expression profiles (Kim *et al.*, 2018; Svensson *et al.*, 2017). The aim of this is to subdivide the cells into separate clusters that represent biologically meaningful sub-populations. Canonical marker genes of known cell types enriched in each cluster are then used to annotate the cluster (consequently all cells within the cluster are given the same label). Despite this being the most common approach, a recent review of clustering algorithms applied to single cell data (Freytag *et al.*, 2018) found little degree of overlap between clusters identified by different methods and their granularity. In part due to the small and variable number of genes detected this results in either under-clustering of the single-cell data or misassignment. Problematically, in both cases, dissimilar cells are grouped together (Freytag *et al.*, 2018; Kim *et al.*, 2018; Shirai *et al.*, 2016).

**Fig. 1:**
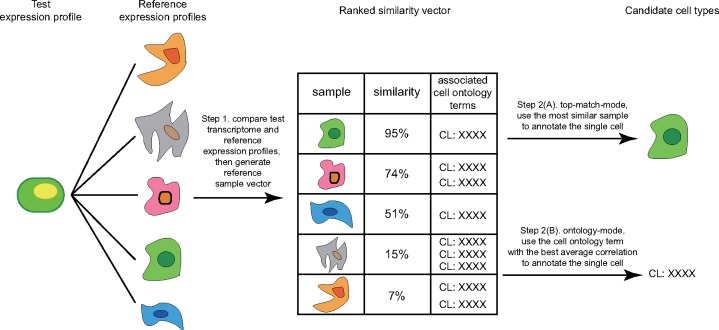
Annotation of single cells using scMatch. scRNA-seq expression profiles are compared against a reference database. Matching samples from the reference database are then ranked by highest to lowest similarity. In top-match mode the cell is annotated with the label of the reference sample with the highest similarity. In ontology-mode, the cell ontology with highest average similarity is used to annotate the query cell. Shapes represent the features of a unique gene expression profile

Here, as an alternative to the cluster-then-annotate approach, we directly annotate single cells without clustering using scMatch, a Python programme that utilizes the similarity between single-cell gene expression profiles and reference expression profiles to directly annotate single cells in a scalable fashion. Basic steps of the annotation pipeline are shown in Figure 1. The first step of the pipeline involves calculating the similarity between gene expression measured in a single cell with reference gene expression profiles from a public database, such as the FANTOM5 atlas [Functional ANnoTation Of Mammalian genomes 5 (Arner *et al.*, 2015; Forrest *et al.*, 2014; Lizio *et al.*, 2017)]. In top-match mode, a cell is simply annotated based on the best correlated sample within the reference database (Fig. 1, Step 2A). Alternatively, ontology-mode uses sample ontology terms (such as the cell ontology; Diehl *et al.*, 2016) to group samples from the same cell type or lineage and then calculates the average correlation (Fig. 1, Step 2B). The ontology term with the highest average correlation is then used to annotate the cell. We benchmark scMatch by evaluating its annotation recall using several public single cell datasets where the identity of every single cell is already known. We show that the choice of correlation measure, the sequencing depth, the cell types in question and the reference database all have an impact on the annotation accuracy. Despite this, scMatch performs well for a broad collection of cell types, and is robust to variations in sequencing depth.

## 2 Materials and methods

### 2.1 Count table down sampling

Existing down-sampling methods usually retain a random subset of the reads in a SAM or BAM file. From the count table, all aligned reads are known. To down-sample reads in a cell to a certain amount, we first calculate the retain probability *P* by dividing the target read count by the original read count. If the target read count is not less than the original read count, then the retain probability is 100%. After gaining the retain probability, we use the *P* to get the subset of detected reads in the cell. If the read count of a gene is *C*, then we randomly draw one or zero *C* times, providing the probability to draw one is *P*. The sum of the draw results is the down-sampled read count of that gene. In this way, approximately *P* × original reads are retained. Since the down-sampling is a stochastic process, the down-sampled count tables with the same retain probability are not identical. We therefore, down-sample a count table ten times and analyse all resulting tables to minimize the technical biases. The annotation recall plotted in the down sampling analysis is the number of correctly annotated single cells in 10 down-sampled count tables divided by the total number of single cells in these 10 tables.

**Table 2.** Annotation recalls of scMatch on four down-sampled cell lines using Spearman’s correlation coefficient

		Spearman	
		Remove zeros (%)	Keep zeros (%)
A549	150 000 reads	99.20	100.00
	100 000 reads	98.00	100.00
	50 000 reads	94.60	100.00
	10 000 reads	87.30	99.70
	1000 reads	63.00	92.20
K562	150 000 reads	95.20	100.00
	100 000 reads	92.90	100.00
	50 000 reads	90.80	100.00
	10 000 reads	85.10	99.50
	1000 reads	14.50	94.80
GM12878 batch1	150 000 reads	80.30	90.00
	100 000 reads	74.70	89.70
	50 000 reads	66.90	90.00
	10 000 reads	41.20	89.10
	1000 reads	14.40	79.70
GM12878 batch2	150 000 reads	99.70	100.00
	100 000 reads	99.60	100.00
	50 000 reads	98.40	100.00
	10 000 reads	92.80	100.00
	1000 reads	46.10	96.60
H1 batch1	150 000 reads	98.30	100.00
	100 000 reads	98.00	100.00
	50 000 reads	97.40	100.00
	10 000 reads	96.20	100.00
	1000 reads	50.00	99.40
H1 batch2	150 000 reads	100.00	100.00
	100 000 reads	100.00	100.00
	50 000 reads	100.00	100.00
	10 000 reads	99.60	100.00
	1000 reads	59.30	99.10

*Note*: The recalls at depths varying from 1000 read up to 150 000 reads calculated (i) only using detected genes or (ii) using all genes. Average recalls of 10 random down-samplings are shown for each. Batch1 and batch2 correspond to biological replicates. Annotation recall is calculated as in Table 1.

### 2.2 Highly expressed and lineage-specific gene lists

Highly expressed and lineage-specific genes were extracted from the FANTOM5 expression atlas. The 4129 highly expressed genes correspond to those detected in the FANTOM5 atlas with maximum expression ≥500 tags per million. The 272 lineage-specific genes were manually curated by examining the expression profiles of genes with maximum expression in the FANTOM5 atlas above 100 tags per million (115 are expressed above 5000 Transcripts Per Million (TPM). Note, the default in scMatch is to use all genes; however users are also able to provide custom gene lists if desired.

### 2.3 Reference datasets used in scMatch and SingleR

Reference gene expression data were collected from FANTOM5, SingleR’s Github repository (https://github.com/dviraran/SingleR) and UCSC Xena Cancer browser (https://xenabrowser.net). For the FANTOM5 data 916 human samples (660 primary cell samples and 256 cancer cell line samples were used) and 821 mouse samples (302 tissue samples, 471 primary cell samples and 48 cancer cell line samples) were prepared as high-quality reference datasets [low read count, low-quality samples were excluded as were samples that could not inform on cell type (e.g. lung, testis)]. Cell ontology terms for the FANTOM5 samples were downloaded from the consortium website and underwent further manual annotation. These are available here https://github.com/forrest-lab/scMatch/tree/master/refDB/FANTOM5. 972 human samples and 1188 mouse samples in SingleR’s reference dataset were extracted from R data files (https://github.com/dviraran/SingleR/tree/master/data). Bulk tumour RNA-seq data for 474 melanoma and 172 glioblastoma samples in The Cancer Genome Atlas (TCGA) were downloaded from the UCSC Xena Cancer browser. Note, we provide several of these reference databases via GitHub but users are also able to use their own custom reference databases. The list of samples used in each analysis is provided in Supplementary Table S9.

### 2.4 Single-cell datasets

Test scRNA-seq data were collected from NCBI GEO (Li *et al.* cell line data GSE81861; Tirosh *et al.* melanoma data GSE72056, and from the 10X Genomics website [peripheral blood mononuclear cells (PBMCs), https://support.10xgenomics.com/single-cell-gene-expression/datasets).

### 2.5 Single-cell data annotation using scMatch and SingleR

The evaluation was conducted on a virtual machine equipped with 16 vCPUs and 48 GB of RAM. We ran scMatch using two correlation measures (Pearson and Spearman coefficients) and three subsets of genes (i) all genes in the FANTOM5 atlas (22 049 genes), (ii) highly expressed genes (detected in the FANTOM5 atlas with maximum expression ≥500 tags per million, 4129 genes) and (iii) manually curated lineage-specific genes from the FANTOM5 atlas (272 genes). The top matched samples returned by scMatch were used to evaluate the annotation recalls. SingleR (version 0.1.0) was run following the authors’ instructions and the fine-tuned results were used to evaluate the annotation recalls.

### 2.6 Software implementation

scMatch is written in Python, it can load gene expression data in CSV files or produced by 10X Genomics platform and then annotate them in parallel using any reference datasets. The resulting outputs are stored in Excel and CSV files.

## 3 Results

### 3.1 Evaluating annotation of high coverage scRNA-seq data from cell lines by comparison to the FANTOM5 reference database

To explore methods for direct annotation of single cell expression profiles using previously published bulk expression datasets as reference databases, we first attempted to annotate single cell expression profiles of cell lines using the FANTOM5 expression atlas as a reference. Using single cell data generated by Li *et al.* (2017) (profiled using the SMARTer Ultra-Low RNA Kit platform) we calculated Spearman and Pearson correlations between single cell expression profiles of four cell lines (A549, K562, GM12878, H1 Human embryonic stem cells) and 1829 bulk expression profiles from the FANTOM5 project (Lizio *et al.*, 2017). We chose to focus on these four cell lines as corresponding bulk expression profiles were available in the FANTOM5 atlas. We considered a single cell correctly annotated if the best correlation corresponded to the matching cell line in FANTOM5 (Table 1). Note for the human embryonic stem cells we considered any match to any human embryonic stem cell lines and human induced pluripotent stem cells in FANTOM5 as a correct annotation. We also took advantage of biological replicates of H1 and GM12878 cells provided in the Li *et al.* paper to examine reproducibility.

**Table 1.** Annotation recalls of scMatch on four deeply profiled cell lines

	Remove zeros		Keep zeros	
	Spearman (%)	Pearson (%)	Spearman (%)	Pearson (%)
A549	100.00	14.90	100.00	45.90
K562	95.90	1.40	100.00	20.50
GM12878 batch1	81.20	0.00	93.80	0.00
GM12878 batch2	100.00	50.00	100.00	57.30
H1 batch1	100.00	82.60	100.00	94.20
H1 batch2	100.00	84.40	100.00	96.90

*Note*: Spearman’s and Pearson’s correlation coefficients were calculated between individual cells from the Li *et al.* (2017) dataset and all FANTOM5 samples, using either all genes (keep zeros), or detected genes only (remove zeros). An annotation was considered correct if the matching cell type in FANTOM5 had the highest correlation. Annotation recall is calculated as the number of cells that were correctly annotated by scMatch divided by the total number of cells of that type in the Li *et al.* dataset. The number of single cells for each cell line is: A549: 74 cells, K562: 73 cells, GM12878 batch1: 32 cells, GM12878 batch12: 96 cells, H1 batch1: 69 cells and H1 batch2: 96 cells. Batch1 and batch2 correspond to biological replicates.

From this comparison we found Spearman’s correlation coefficient outperformed Pearson’s correlation for all six libraries. Specifically, the annotation recalls for A549, K562, GM12878 (Batch 2) and the two H1 human embryonic stem cell batches were 100%, while that for GM12878 (Batch 1) was 93.75% (30 cells out of 32) (Table 1, Supplementary Table S1). We also carried out a parallel analysis where the correlations were only calculated using the genes detected in each cell (i.e. genes detected in FANTOM5 but not in the query cell were not used in the calculation of the correlation). However using all genes and Spearman’s correlation coefficient provided the best recalls (Table 1).

### 3.2 Impact of sequencing depth on annotation recall

In the above analysis, the median number of reads per cell was 2 029 222. Our method achieved a high recall on this high sequencing depth data; however, drop-seq-based experimental techniques typically sequence single cells at much lower depths. For example, 10× genomics recommend 20–50 000 reads per cell and Pollen *et al.* have reported that 50 000 reads per cell is sufficient to annotate cells correctly using expressed canonical marker genes (Pollen *et al.*, 2014). We, therefore, down-sampled the Li *et al.* cell line data used above to various fixed read depths per cell, to explore how the sequencing depth affected the annotation (Section 2).

As expected, cells with more reads were more likely to be correctly classified than those down-sampled to lower read depths (Table 2, Supplementary Fig. S1 and Supplementary Table S2). As in Table 1, using all genes (rather than filtering out genes with zero values) yielded the highest recalls. This effect was more apparent at lower reads depths, e.g. when GM12878 Batch 1 was down-sampled to 1000 reads the recall using all genes was 79% but when zeros were filtered out this dropped to 14%. Notably, when zeros were kept, the other five libraries still had high recalls ranging from 92 to 99% at this lowest depth of 1000 reads per cell. In terms of misclassifications, these typically were of closely related cells. For example, single cells from Batch 1 of the lymphoblastoid B cell line GM12878 were sometimes misannotated as primary B cells or other B cell lines (Supplementary Table S3). Last, decreasing the sequencing depth affected recall of different cell types to different degrees reflecting the different ranges of correlations observed for each cell type and differences in the number of similar (but distinct) cell types in the reference database. This has important implications for cell classification. Both sequencing depth and the cell type considered affect the classification performance.

### 3.3. Annotation of PBMCs profiled using the 10X genomics platform

Next to evaluate scMatch on authentic low-depth data, we assessed the performance of our method on a low-sequencing depth dataset obtained from 10 bead purified sub-populations of PBMCs profiled using the 10X genomics chromium 3’ assay (Zheng *et al.*, 2017). In this dataset, each cell was sequenced to a depth of ∼20 000 raw reads which translates to medians of 525 genes and 1300 unique molecular identifier counts per cell.

To determine whether the classification accuracy of scMatch could be improved by using different subsets of genes, we compared the annotations using correlations calculated using (i) All reference genes (22 049 genes) to those obtained using either (ii) Highly expressed genes (detected in the FANTOM5 atlas with maximum expression ≥ 500 tags per million, 4129 genes) or (iii) Manually curated lineage-specific genes from the FANTOM5 atlas (272 genes). To score the annotation accuracy using each gene list we first attempted to map the ten PBMC sub-populations studied in the original publication to their corresponding primary cell types in the FANTOM5 reference dataset (445 primary cell samples, Supplementary Table S9). For all except CD8+CD45RA+ Naïve cytotoxic T cells we were able to find a corresponding sample in FANTOM5 (Supplementary Table S4).

For four of the nine PBMC sub-populations with matching samples in FANTOM5, cell annotation using Spearman’s correlation coefficients yielded the best recalls; CD19+ B cells, CD8+ cytotoxic T cells, CD14+ monocytes were best annotated using all genes, while CD34+ haematopoietic stem cells were equally well annotated using all genes or using only highly expressed genes (from Table 3 and Supplementary Table S5). For the other five sub-populations (T cell subsets and NK cells) Pearson’s correlation coefficients, with various gene lists, yielded better recalls. In particular CD4+ helper T cells, CD4+/CD45RA+/CD25− Naïve T cells and CD4+/CD25+ Regulatory T cells had recalls above 50% using either all genes or highly expressed genes. In contrast CD56+ NK cells and CD4+/CD45RO+ Memory T cells had poor recall regardless of the correlation method or the gene sets used.

**Table 3.** Annotation of 93 655 PBMC cells profiled on the 10X platform

	Specific genes		Highly expressed genes		All genes	
	Pearson (%)	Spearman (%)	Pearson (%)	Spearman (%)	Pearson (%)	Spearman (%)
CD4+/CD25+ regulatory T cells	37.7	8.4	55.6	53.2	59.7	19.3
CD8+ cytotoxic T cells	30.7	37.5	2.0	76.4	2.0	98.3
CD19+ B cells	92.0	41.5	38.1	99.9	37.4	100.0
CD56+ NK cells	0.6	4.9	22.9	0.0	20.6	0.0
CD4+ helper T cells	55.5	68.1	98.2	30.0	98.4	5.8
CD14+ monocytes	10.7	78.8	28.0	42.9	27.5	78.8
CD34+ cells	0.8	4.4	56.3	83.0	36.0	82.8
CD4+/CD45RO+ memory T cells	44.0	0.5	26.3	12.7	28.5	5.4
CD4+/CD45RA+/CD25− naive T cells	11.1	51.0	86.6	5.5	85.9	7.7
CD8+/CD45RA+ naive cytotoxic T cells	29.7	35.8	0.0	86.5	0.0	99.1

*Note*: The relative recalls for PBMCs when annotated using scMatch with the FANTOM5 dataset as a reference. (i) Pearson’s correlation coefficient and (ii) Spearman’s correlation. Accuracy calculated using different gene lists is shown [(i) All reference set genes (22 049 genes), (ii) Highly expressed genes (detected in the FANTOM5 atlas with maximum expression ≥500 tags per million, 4129 genes) and (iii) Manually curated lineage-specific genes from the FANTOM5 atlas (272 genes)]. Annotation recall is calculated as the number of cells that were correctly annotated by scMatch divided by the total number of cells of that type in the Zheng *et al.* (2017) dataset.

Next we evaluated the use of the ‘ontology-mode’ (using Spearman’s correlations on all genes, Fig. 1) to annotate this dataset. To do this we used the cell ontology (CL) annotations provided in the FANTOM5 atlas. For each single cell, correlations against reference samples in FANTOM5 with the same cell ontology annotation were averaged, and then cell ontology matches were sorted based on the average correlation. The results for Spearman correlations are summarized in Supplementary Table S6. As expected, for cell types where we already had high recall their annotations did not dramatically change. For example, almost 100% of B-cells were annotated as CL: 0000236: B cell, and 83% of CD34+ cells were annotated as CL: 0000037: hematopoietic stem cell. However, for multiple cell types the annotation recalls improved in ontology-mode. E.g. CD14+ monocytes achieved a recall of 78.8% in top-match mode but 94.3% were annotated as CL: 0002397: CD14-positive, CD16-positive monocyte in ontology mode, similarly CD4+/CD45RO+ Memory T cells only achieved a recall of 5.4% in top-match mode, but 45.9% were correctly annotated as CL: 0002678: memory regulatory T cell in ontology mode while the remainder mapped to other CD4+ subsets (30% were annotated as CL: 0002677: naive regulatory T cell and 21% as CL: 0000897: CD4-positive, alpha-beta memory T cell) (Supplementary Table S6). We envisage ‘ontology-mode’ will be useful to give an indication of the cell lineage when a cell type that is not in the reference database needs to be annotated.

### 3.4 Performance of scMatch and SingleR using identical reference databases

Recently Aran *et al.* (2019) presented an R package SingleR similar in concept to scMatch. For human cell annotation, SingleR uses 259 bulk RNA-seq samples from the BLUEPRINT (Fernandez *et al.*, 2016) and ENCODE (Sloan *et al.*, 2016) projects and 713 bulk microarray samples from the Human Primary Cell Atlas (HPCA) (Mabbott *et al.*, 2013) as reference expression data. To compare the performance of scMatch (Spearman’s correlation, all genes) to SingleR we annotated all cells from the 10X PBMC dataset above using both methods with either (i) BLUEPRINT + ENCODE, (ii) HPCA, (iii) BLUEPRINT + ENCODE + HPCA or (iv) BLUEPRINT + ENCODE + HPCA + FANTOM5 as reference databases (see Table 4, Supplementary Table S6).

**Table 4.** The recalls of annotating the PBMCs using scMatch and SingleR using ENCODE, BLUEPRINT and HPCA datasets

	BLUEPRINT+ENCODE		HPCA		BLUEPRINT+ENCODE +HPCA		BLUEPRINT+ENCODE +HPCA+FANTOM5
	SingleR (%)	scMatch (%)	SingleR (%)	scMatch (%)	SingleR (%)	scMatch (%)	scMatch (%)
CD4+/CD25+ Regulatory T cells	42.8	28.1	0.0	0.0	43.1	37.9	39.0
CD8+ Cytotoxic T cells	82.7	27.5	18.0	8.9	34.0	43.0	44.8
CD19+ B cells	99.9	66.5	100.0	99.9	90.5	99.0	99.2
CD56+ NK cells	99.2	95.8	95.1	93.3	95.0	98.4	98.5
CD4+ Helper T cells	94.5	86.4	99.5	99.5	93.5	98.2	97.9
CD14+ Monocytes	98.1	95.5	83.2	95.6	96.4	97.6	97.7
CD34+ cells	84.6	88.2	80.4	85.3	88.5	85.7	85.3
CD4+/CD45RO+ Memory T cells	75.4	80.8	95.3	97.5	73.1	86.8	86.0
CD4+/CD45RA+/CD25− Naive T cells	N/A	N/A	85.3	85.3	0.0	0.0	0.0
CD8+/CD45RA+ Naive Cytotoxic T cells	N/A	N/A	0.0	0.0	0.0	0.0	0.0

*Note*: Comparison of scMatch and SingleR using different reference datasets. Annotation recall is calculated as in Table 3. (i) We used SingleR in fine-tuning mode, (ii) There are no matched reference samples in the BLUEPRINT + ENCODE database for CD4+/CD45RA+/CD25− Naïve T cells and CD8+/CD45RA+ Naïve cytotoxic T cells.

For the majority of cell types SingleR and scMatch had similar accuracy; however, the relative accuracy of each depended greatly on the reference database used (Table 4). For example, when using the ENCODE + BLUEPRINT RNA-seq data as reference, SingleR outperformed scMatch in six out of eight comparisons. When using HPCA as the reference SingleR had better recall twice, scMatch had better recall three times and there were three ties (defined as <0.5% difference). Notably no regulatory T cells were correctly annotated when using the HPCA as reference. Last when using the combined BLUEPRINT+ENCODE+HPCA dataset as reference, scMatch outperformed SingleR six times, SingleR outperformed scMatch twice, and neither were able to correctly annotate the two Naïve T cell populations (these typically matched to bulk CD4 or CD8, respectively).

To demonstrate scMatch’s usage on a large reference dataset, we next merged BLUEPRINT+ENCODE+HPCA and FANTOM5 into a single reference dataset of 1417 samples and used it to annotate the PBMCs. For six of the cell types the annotations obtained by scMatch using BLUEPRINT+ENCODE+HPCA+FANTOM5 and BLUEPRINT+ENCODE+HPCA were comparable (<0.5% difference) however for the CD4+/CD25+ Regulatory T cells and the CD8+ Cytotoxic T cells there were increases in recall of 1.07 and 1.81%, respectively (Table 4). We also examined the impact of increasing the number of reference samples on false positive rates and found that the higher recalls observed with more reference samples were accompanied with high precision (Supplementary Table S7). Thus incorporating more reference samples improved the performance of scMatch.

In terms of run times. When we used BLUEPRINT+ENCODE+ HPCA as the reference dataset, on virtual machines with identical resources (16 vCPUs and 48 GB of RAM), scMatch was able to use up to 10 CPUs and took 40.5 h to annotate all 93 655 PBMCs while SingleR was only able to utilize at most two CPUs and took 193 h. This is largely because scMatch uses memory more efficiently. This may also explain why we were unable to load the combined BLUEPRINT+ENCODE+HPCA+ FANTOM5 dataset into SingleR to assess its performance.

### 3.5 Application of scMatch to cancer datasets

Previously, Tirosh *et al.* (2016) deeply sequenced 4645 single cells from 19 human melanoma tumours of various clinical and therapeutic backgrounds and detected the identities of these cells through flow cytometry, genetic and transcriptional profiles. Using scMatch and Spearman correlations we next annotated this dataset by comparison against the 916 FANTOM5 primary cells and cell lines, which include two melanoma cell lines (G-361 and COLO 679) and primary melanocytes. As shown in Supplementary Table S8, 94% of the cells (1187/1257) originally annotated as melanoma ‘malignant cells’ were annotated as either melanoma cells (31%) or melanocytes (63%).

As a key step in single-cell analysis of tumour samples is the classification of cells as tumour or normal cells, we next assessed whether expansion of the reference database in scMatch by incorporating bulk RNA-seq datasets from TCGA) (Cancer Genome Atlas Network, 2015) would improve the discrimination of melanoma cells from melanocytes. To do this we added 474 bulk melanoma profiles and 172 bulk glioblastoma profiles (as an unrelated tumour type) to the FANTOM5 reference database (Supplementary Table S9).

By adding these two bulk RNA-seq datasets and rerunning scMatch against this extended reference dataset, the majority of melanoma cells were now correctly annotated as melanoma (83.29%, 1047 cells out of 1257). We note only three cells in the dataset were now misannotated as glioblastoma (Supplementary Table S8). Additionally, three B cells and 36 T cells were now annotated as melanoma. Examining the scMatch results for these lymphocytes revealed they most closely matched melanoma lymph node metastases which are likely to contain large fractions of lymphocytes.

Last, we investigated the expression of melanocytic markers in cells that were annotated as ‘malignant cells’, ‘non-malignant cells’ or ‘unresolved’ (based on Tirosh *et al.*’s inferred Copy Number Variation (CNV) and marker gene analyses from the original publication) and compared these to ‘melanoma’, ‘melanocyte’ and ‘other cell type’ annotations provided by scMatch (Supplementary Fig. S2). As expected, the majority of cells annotated by both Tirosh *et al.* as ‘malignant cells’ and by scMatch as melanocytic expressed high levels of the melanocytic markers *MITF, PMEL, MLANA* and *TYR*. Additionally 90 of the 132 cells labelled as ‘unresolved’ in the original publication, and 86 of the 416 cells labelled as ‘non-malignant cells’ were classified as melanoma cells or melanocytes by scMatch, the majority of which expressed melanocytic markers. Last, 14 cells classified as ‘malignant cells’ in the original publication were annotated as other cell types by scMatch. These are potentially misannotations in the original publication as they do not express *TYR* or *MITF* and only 1 expresses *PMEL* (Supplementary Fig. S2), but we cannot rule out dropout or mutations in these cells that inactivate these melanocytic markers. Together, this suggests that scMatch will be useful for annotation of tumour cells within single-cell datasets and that the power to correctly classify these cells may be improved by selective leveraging of large bulk tumour datasets such as TCGA.

## 4 Discussion

scRNA-seq is a rapidly evolving and increasingly popular technique for transcriptomic analysis of complex tissues. To reach its full potential, a reliable approach for cell type classification is required. Most contemporary approaches employ unsupervised clustering of cell expression profiles, followed by manual annotation of each cluster’s identity based on gene expression markers (Kim *et al.*, 2018; Svensson *et al.*, 2017). However, the unsupervised clustering algorithms are known to yield inconsistent results (Freytag *et al.*, 2018). Additionally, manual cluster annotation is subjective, non-scalable and non-reproducible. To overcome these limitations, we developed scMatch, a marker-free annotation programme for scRNA-seq data based on a test-to-reference per cell comparison.

In development of scMatch, we first systematically compared the accuracy of the cell annotations using different similarity measures (Pearson’s versus Spearman’s correlation coefficients), different gene lists (all genes, cell type-specific genes and highly expressed genes) and with single-cell data at different sequencing depths. In most cases, Spearman’s rank correlation and using all genes yielded higher recall rates (Tables 1 and 3) and thus we recommend this option to users, red however for flexibility, we provide the user the options of using Pearson’s correlation and different user-defined sets of genes. To assess the impact of sequencing depth on the annotation recall, we performed cell type annotation of down-sampled human cell line data. As might be expected, annotation recall rates decreased with lower read depths; however, even when the depth was down-sampled to as low as 1000 reads, the recall rates were still high at 79–99% making scMatch a useful tool for annotating low-depth single-cell experiments (Table 2).

scMatch is not the first tool to compare single cells to reference datasets. SingleR, recently introduced by Aran *et al.* uses a similar approach; however, scMatch has some advantages over SingleR. When using all genes (the best performing and recommended mode for scMatch) no pre-processing of test and reference gene expression data is required. It is also computationally more efficient as (i) the reference dataset is held in a simpler data structure which requires less memory and (ii) it does not incorporate a computationally intensive fine-tuning step. In terms of accuracy, the results were comparable between scMatch and SingleR (Table 4). However, when using the combined reference dataset of BLUEPRINT+ENCODE+HPCA, scMatch had higher recall than SingleR for 6 out of 10 PBMC cell types tested. A possible explanation as to why this occurs may be because SingleR calculates correlations only using the variable genes in the reference dataset. By combining samples from multiple reference datasets the set of variable genes identified by SingleR may change and thus affect the annotations. In contrast scMatch uses all genes as default, thus adding extra samples will not affect the correlations of existing samples. Additionally, scMatch offers ontology-mode which should be useful in cases where a matching cell type is absent but knowing cell lineage is still informative. Last, scMatch reports the sample in the reference dataset with the highest correlation, however these correlations vary with read depth in each cell and cell type considered (Supplementary Fig. S1). Currently we are unable to provide a meaningful confidence measure for annotations reported by scMatch. Cell types not present in the reference dataset will still be annotated with their closest match and closely related subtypes (e.g. T cells) may be difficult to discriminate.

For these methods the reference dataset used has a large impact. For example, when the FANTOM5 primary cell dataset was used as a reference, natural killer cells were poorly annotated (Table 3), however, when the HPCA and BLUEPRINT+ENCODE were added, the recall approached 99% (Table 4). This was also associated with high precision (Supplementary Table S7). Similarly when we extended the reference dataset to include bulk tumour data from the TCGA the annotation of melanoma cells improved (Supplementary Fig. S2). We note however there are several considerations in regards to the reference dataset, including (i) the relative representation of each cell type, (ii) the desired granularity of cell types represented, (iii) the quality of the reference samples and (iv) the inclusion of reference datasets generated using different platform technologies.

In principle, the addition of more reference samples for the same cell type should provide a better representation of the diversity within a cell type, but it also has the potential to increase the number of cells hitting this cell type by chance. When annotating PBMCs using the FANTOM5 references we saw no relationship between the total number of matched reference samples and recall rates (Supplementary Table S5). For example for CD4+ helper T cells, there were 26 reference samples but only a recall rate of 5.75% (Spearman all genes). In contrast, the CD34+ stem cells which only had five reference samples had a recall of 82.83%. Importantly adding a large number of reference samples corresponding to a cell type absent from the test dataset (172 bulk glioblastoma RNA-seq samples) introduced very few mis-annotations with only 3 of the 4645 cells in the melanoma dataset, mis-annotated as glioblastoma. We note, although CAGE, RNA-seq and microarrays have different dynamic ranges and biases, the inclusion of reference datasets measured on these different platforms improved the recall rates for both the PBMCs and the melanoma dataset. We attribute this to the meta-dataset containing a better coverage of cell states than any of the single datasets alone. Thus for the novice user we recommend to use all available relevant databases. Specifically, for normal tissues and primary cells we recommend using all available primary cell data, while for tumour samples inclusion of cell lines and bulk tumour samples is recommended to identify tumour cells.

In conclusion, with the availability of large numbers of single cell datasets over the coming years, driven by the Human Cell Atlas (Regev *et al.*, 2018) and others (Han *et al.*, 2018; Tabula Muris *et al.*, 2018), scalable methods such as scMatch are needed for cell classification. Conversely, the cell types identified and their expression profiles in these atlases can be fed in as reference datasets to improve the annotation of future experiments.

## Funding

This work was carried out with the support of a collaborative cancer research grant provided by the Cancer Research Trust ‘Enabling advanced single-cell cancer genomics in Western Australia’ and a grant from the Cancer Council of Western Australia. R.H. is supported by an Australian Government Research Training Programme (RTP) Scholarship. A.F. was supported by funds raised by the MACA Ride to Conquer Cancer and a Senior Cancer Research Fellowship from the Cancer Research Trust. A.F. is currently supported by an Australian National Health and Medical Research Council Fellowship [APP1154524]. Analysis was made possible with computational resources provided by the Pawsey Supercomputing Centre with funding from the Australian Government and the Government of Western Australia.

### Author contributions

R.H. implemented scMatch in python, carried out all analyses and generated all figures and tables. A.R.R.F. designed the study. R.H., E.D. and A.R.R.F. wrote the article. All authors have read and approved of the final version of the article.

*Conflict of Interest*: none declared.

## Supplementary Material

btz292_Supplementary_Materials

## References

[btz292-B1] Aran D. et al (2019) Reference-based analysis of lung single-cell sequencing reveals a transitional profibrotic macrophage. Nat. Immunol., 20, 163–172. 30643263 10.1038/s41590-018-0276-y PMC6340744

[btz292-B2] Arner E. et al (2015) Transcribed enhancers lead waves of coordinated transcription in transitioning mammalian cells. Science, 347, 1010–1014. 25678556 10.1126/science.1259418 PMC4681433

[btz292-B3] Bendall S.C. et al (2014) Single-cell trajectory detection uncovers progression and regulatory coordination in human B cell development. Cell, 157, 714–725. 24766814 10.1016/j.cell.2014.04.005 PMC4045247

[btz292-B4] Cancer Genome Atlas Network. (2015) Genomic classification of cutaneous melanoma. Cell, 161, 1681–1696. 26091043 10.1016/j.cell.2015.05.044 PMC4580370

[btz292-B5] da Rocha E.L. et al (2018) Reconstruction of complex single-cell trajectories using CellRouter. Nat. Commun., 9, 892. 29497036 10.1038/s41467-018-03214-y PMC5832860

[btz292-B6] Diehl A.D. et al (2016) The Cell Ontology 2016: enhanced content, modularization, and ontology interoperability. J. Biomed. Semantics, 7, 44. 27377652 10.1186/s13326-016-0088-7 PMC4932724

[btz292-B7] Fernandez J.M. et al (2016) The BLUEPRINT data analysis portal. Cell Syst., 3, 491. 27863955 10.1016/j.cels.2016.10.021 PMC5919098

[btz292-B8] Forrest A.R. et al (2014) A promoter-level mammalian expression atlas. Nature, 507, 462–470. 24670764 10.1038/nature13182 PMC4529748

[btz292-B9] Freytag S. et al (2018) Comparison of clustering tools in R for medium-sized 10x Genomics single-cell RNA-sequencing data. F1000Res., 7, 1297. (30228881 10.12688/f1000research.15809.1 PMC6124389

[btz292-B10] Haghverdi L. et al (2016) Diffusion pseudotime robustly reconstructs lineage branching. Nat. Methods, 13, 845. 27571553 10.1038/nmeth.3971

[btz292-B11] Han X.P. et al (2018) Mapping the mouse cell atlas by Microwell-seq. Cell, 172, 1091. +. 29474909 10.1016/j.cell.2018.02.001

[btz292-B12] Hashimshony T. et al (2012) CEL-Seq: single-cell RNA-seq by multiplexed linear amplification. Cell Rep., 2, 666–673. 22939981 10.1016/j.celrep.2012.08.003

[btz292-B13] Kim T. et al (2018) Impact of similarity metrics on single-cell RNA-seq data clustering. Brief. Bioinform., doi: 10.1093/bib/bby076. 10.1093/bib/bby076 30137247

[btz292-B14] Klein A.M. et al (2015) Droplet barcoding for single-cell transcriptomics applied to embryonic stem cells. Cell, 161, 1187–1201. 26000487 10.1016/j.cell.2015.04.044 PMC4441768

[btz292-B15] Li H. et al (2017) Reference component analysis of single-cell transcriptomes elucidates cellular heterogeneity in human colorectal tumors. Nat. Genet., 49, 708–718. 28319088 10.1038/ng.3818

[btz292-B16] Lizio M. et al (2017) Update of the FANTOM web resource: high resolution transcriptome of diverse cell types in mammals. Nucleic Acids Res., 45, D737–D743. 27794045 10.1093/nar/gkw995 PMC5210666

[btz292-B17] Mabbott N.A. et al (2013) An expression atlas of human primary cells: inference of gene function from coexpression networks. BMC Genomics, 14, 632. 24053356 10.1186/1471-2164-14-632 PMC3849585

[btz292-B18] Macosko E.Z. et al (2015) Highly parallel genome-wide expression profiling of individual cells using nanoliter droplets. Cell, 161, 1202–1214. 26000488 10.1016/j.cell.2015.05.002 PMC4481139

[btz292-B19] Picelli S. et al (2013) Smart-seq2 for sensitive full-length transcriptome profiling in single cells. Nat. Methods, 10, 1096–1098. 24056875 10.1038/nmeth.2639

[btz292-B20] Pollen A.A. et al (2014) Low-coverage single-cell mRNA sequencing reveals cellular heterogeneity and activated signaling pathways in developing cerebral cortex. Nat. Biotechnol., 32, 1053. +. 25086649 10.1038/nbt.2967 PMC4191988

[btz292-B21] Regev A. et al (2018) The Human Cell Atlas White Paper. arXiv Preprint arXiv, 05192, 2018.

[btz292-B22] Rosenberg A.B. et al (2018) Single-cell profiling of the developing mouse brain and spinal cord with split-pool barcoding. Science, 360, 176. 29545511 10.1126/science.aam8999 PMC7643870

[btz292-B23] Setty M. et al (2016) Wishbone identifies bifurcating developmental trajectories from single-cell data. Nat. Biotechnol., 34, 637–645. 27136076 10.1038/nbt.3569 PMC4900897

[btz292-B24] Shin J. et al (2015) Single-cell RNA-seq with waterfall reveals molecular cascades underlying adult neurogenesis. Cell Stem Cell, 17, 360–372. 26299571 10.1016/j.stem.2015.07.013 PMC8638014

[btz292-B25] Shirai M. et al (2016) Vertical flow array chips reliably identify cell types from single-cell mRNA sequencing experiments. Sci. Rep., 6, 36014. 27876759 10.1038/srep36014 PMC5120284

[btz292-B26] Sloan C.A. et al (2016) ENCODE data at the ENCODE portal. Nucleic Acids Res., 44, D726–D732. 26527727 10.1093/nar/gkv1160 PMC4702836

[btz292-B27] Svensson V. et al (2017) Power analysis of single-cell RNA-sequencing experiments. Nat. Methods, 14, 381. +. 28263961 10.1038/nmeth.4220 PMC5376499

[btz292-B28] Tabula Muris C. et al (2018) Single-cell transcriptomics of 20 mouse organs creates a Tabula Muris. Nature, 562, 367–372. 30283141 10.1038/s41586-018-0590-4 PMC6642641

[btz292-B29] Tang F.C. et al (2009) mRNA-Seq whole-transcriptome analysis of a single cell. Nat. Methods, 6, 377–386. 19349980 10.1038/nmeth.1315

[btz292-B30] Tay S. et al (2010) Single-cell NF-kappa B dynamics reveal digital activation and analogue information processing. Nature, 466, 267–271. 20581820 10.1038/nature09145 PMC3105528

[btz292-B31] Thompson A.M. et al (2014) Self-digitization microfluidic chip for absolute quantification of mRNA in single cells. Anal. Chem., 86, 12308–12314. 25390242 10.1021/ac5035924 PMC4270397

[btz292-B32] Tirosh I. et al (2016) Dissecting the multicellular ecosystem of metastatic melanoma by single-cell RNA-seq. Science, 352, 189–196. 27124452 10.1126/science.aad0501 PMC4944528

[btz292-B33] Trapnell C. et al (2014) The dynamics and regulators of cell fate decisions are revealed by pseudotemporal ordering of single cells. Nat. Biotechnol., 32, 381–386. 24658644 10.1038/nbt.2859 PMC4122333

[btz292-B34] Treutlein B. et al (2014) Reconstructing lineage hierarchies of the distal lung epithelium using single-cell RNA-seq. Nature, 509, 371–375. 24739965 10.1038/nature13173 PMC4145853

[btz292-B35] Wang B.L. et al (2014) Microfluidic high-throughput culturing of single cells for selection based on extracellular metabolite production or consumption. Nat. Biotechnol., 32, 473–478. 24705516 10.1038/nbt.2857 PMC4412259

[btz292-B36] Zheng G.X.Y. et al (2017) Massively parallel digital transcriptional profiling of single cells. Nat. Commun., 8, 14049. 28091601 10.1038/ncomms14049 PMC5241818

