# Supplementary material for: scMatch: a single-cell gene expression profile annotation tool using reference datasets
Source: Bioinformatics. 2019 Apr 26;35(22):4688–95. doi: 10.1093/bioinformatics/btz292 (PMC6853649; doi:10.1093/bioinformatics/btz292)
Supplement: btz292_Supplementary_Materials [file btz292_supplementary_materials.zip › btz292-suppl_data/Supplementary Materials.doc]

**Supplementary Tables:**

**Supplementary Table 1. Annotation recalls of scMatch on six deeply profiled cell lines.** Using Pearson's correlation coefficients and Spearman's correlation coefficients.

**Supplementary Table 2. Annotation recalls of scMatch on six down-sampled cell lines using Spearman's correlation coefficients.**

**Supplementary Table 3. The top annotations provided by scMatch on the down-sampled data used to compute the annotation recalls shown in Supplementary Table 2.**

**Supplementary Table 4. PBMC samples in the FANTOM5, HPCA, ENCODE and Blueprint datasets considered as matches for the 10 PBMC populations from Zheng *et al*.**

**Supplementary Table 5. Annotation recalls of the PBMCs using Pearson's or Spearman's correlation coefficients on different gene lists with FANTOM5 dataset.**

**Supplementary Table 6. Annotation results of the PBMCs using scMatch and SingleR with ENCODE, BLUEPRINT, HPCA, and FANTOM5 as reference datasets (columns are ten PBMC sub-populations, rows are the number of annotations in PBMCs).**

**Supplementary Table 7. Annotation recall and precision of the six PBMC subpopulations using scMatch with ENCODE, BLUEPRINT, HPCA, and FANTOM5 as reference datasets.**

**Supplementary Table 8. Annotation of cells in the Tirosh *et al.* melanoma dataset using scMatch with FANTOM5, bulk melanoma and glioblastoma samples in TCGA as reference datasets.**

**Supplementary Table 9. The reference samples used in each analysis in this paper.**

**Supplementary Figures:**

**Supplementary Figure 1. Features of accurately annotated cells using Pearson's correlation coefficients (a-c) and Spearman's correlation coefficients separately (d-f) with all genes in the FANTOM5 dataset. a)** and **d)** The distribution of total read depth per cell for correctly and incorrectly annotated single cells. **b)** and **e)** The distribution of the number of detected genes per cell for correctly and incorrectly annotated single cells**. c)** and **f)** The distribution of the Spearman's correlation coefficients for correctly and incorrectly annotated single cells.

**Supplementary Figure 2. Annotation of cells in the Tirosh *et al.* melanoma dataset.** The plots show expression (log10-transformed TPM values) of four melanocytic marker genes in single cells annotated as either (**a,b**) ‘malignant cells’, (**c,d**) ‘non-malignant cells’ or (**e,f**) unresolved in the Tirosh *et al.* paper. These are compared to the annotations from scMatch ‘melanoma’, ‘melanocyte’ or ‘other’ using either the (**a,c,e**) FANTOM5 dataset alone of the (**b,d,f**) FANTOM5 atlas supplemented with the melanoma samples and glioblastoma samples in TCGA dataset.
